# Supplementary material for: Anti-cancer compound screening identifies Aurora Kinase A inhibition as a means to favor CRISPR/Cas9 gene correction over knock-out
Source: PLoS One. 2025 Sep 26;20(9):e0332617. doi: 10.1371/journal.pone.0332617 (PMC12469161; doi:10.1371/journal.pone.0332617)
Supplement: S1 File — (DOCX) [file pone.0332617.s001.docx]

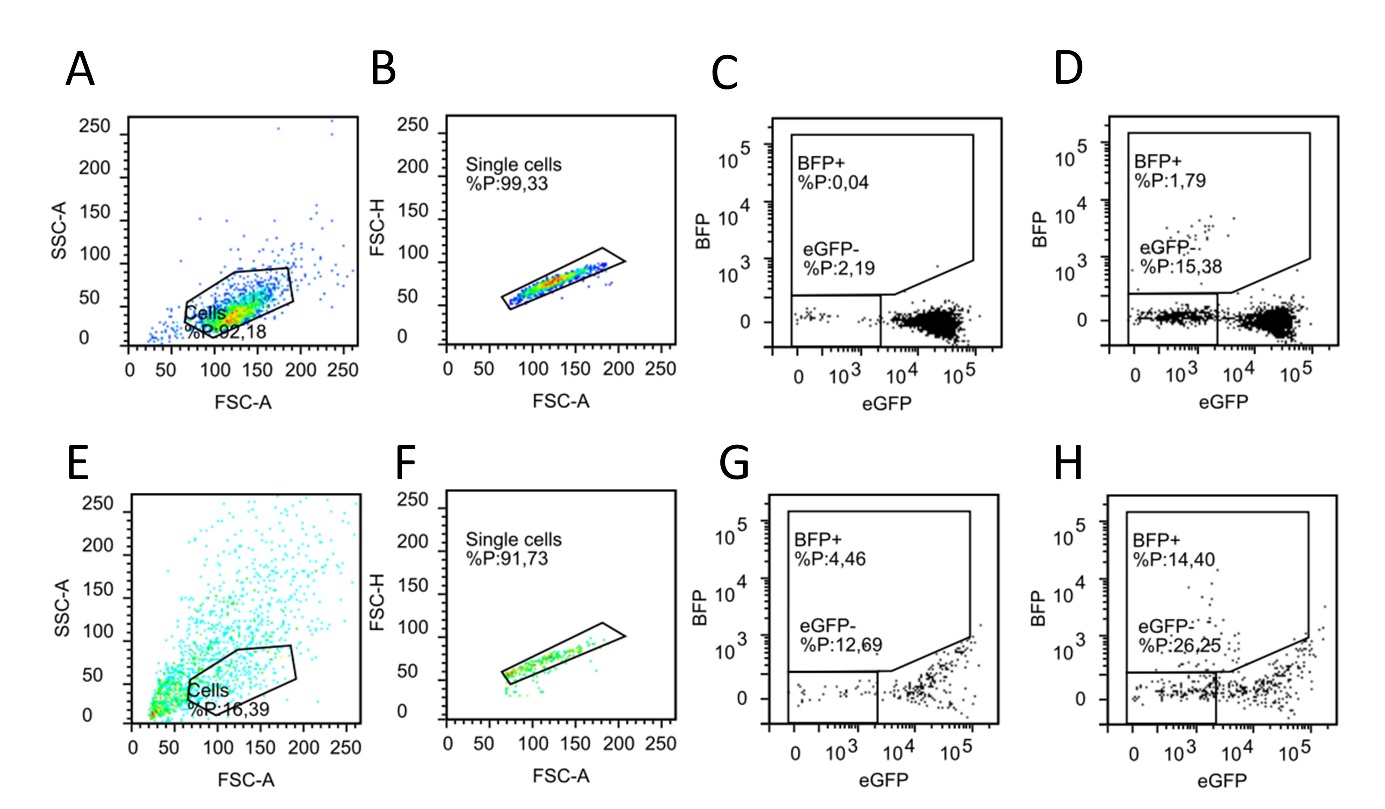


**S1 Figure: Gating strategy employed in typical flow cytometry measurements for gene editing outcomes in HEK293T-eGFP cells.** A-D: Gating without additional compounds. E-H: Gating in presence of 1 µM alisertib. A and E: gating cells. In presence of alisertib the spread of forward and side scatter changes, it is assumed that cells with normal morphology, as seen in figure 3E, are in the same location in this dot plot. B and F: single cell gating. C and G: eGFP knock-out and BFP emergence in absence of gene editing LNP. D and H: eGFP knock-out and BFP emergence in presence of gene editing LNP. NHEJ incidence was calculated in the eGFP- gate in D or H and subtracting the eGFP- gate from C or G respectively. Absolute HDR incidence was calculated in the BFP+ gate in D or H and subtracting the BFP+ gate from C or G, respectively. Relative HDR incidence was calculated by dividing the absolute HDR incidence by the sum of NHEJ incidence and absolute HDR incidence.Notably, alisertib treated cells had a high “false positive” rate in the eGFP- and HDR gates in the control (G), which were subtracted from relevant results.


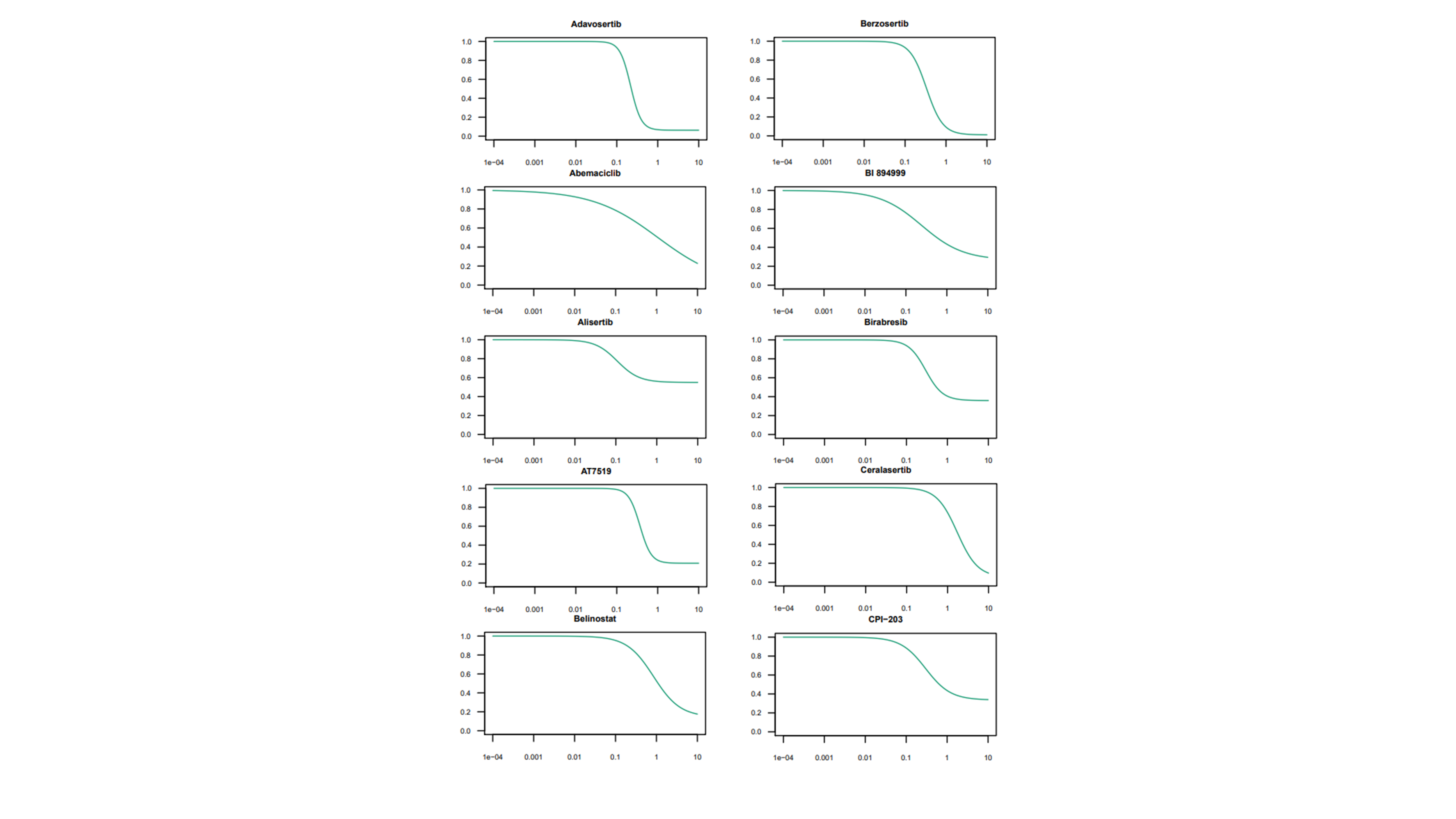


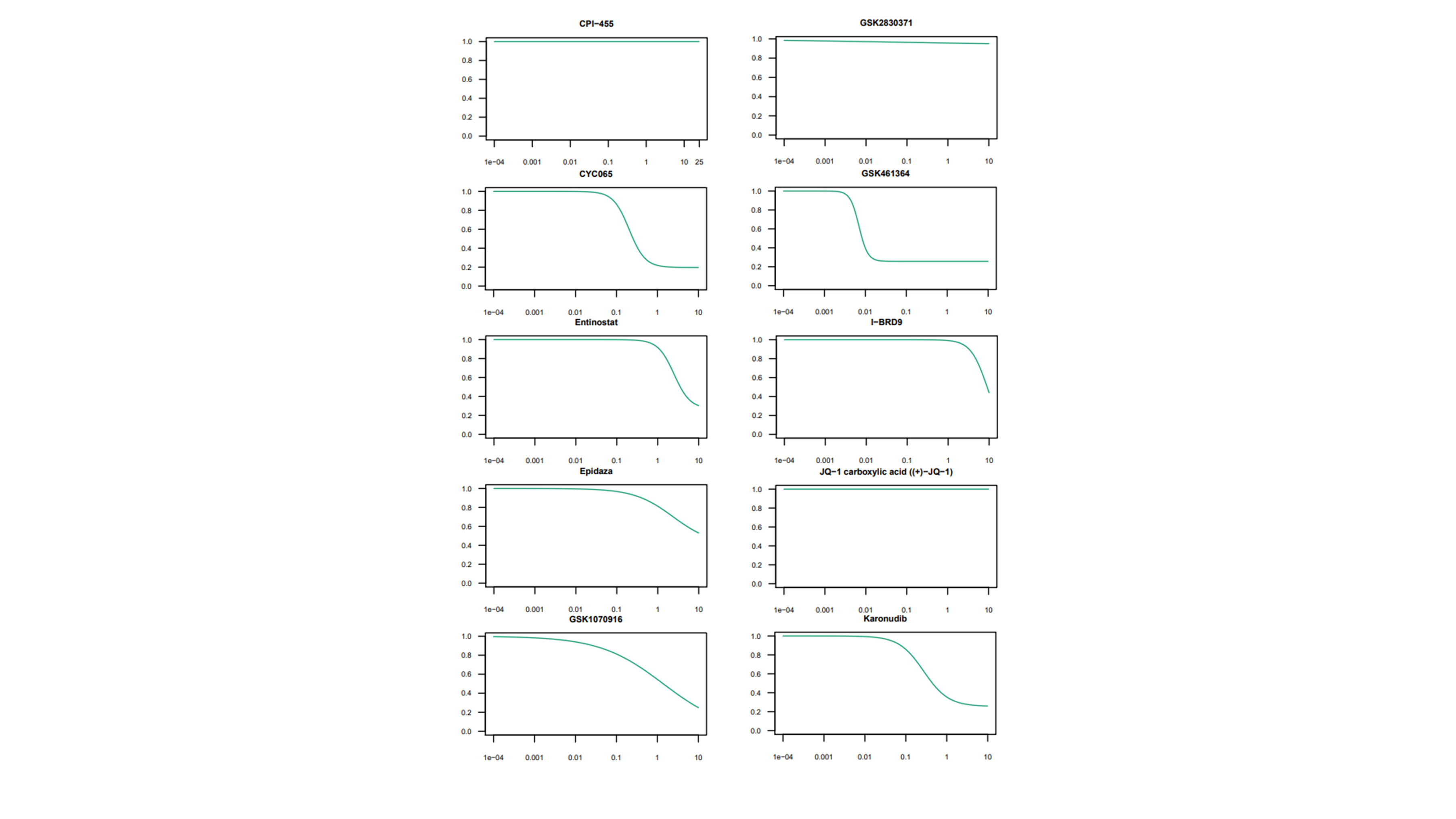


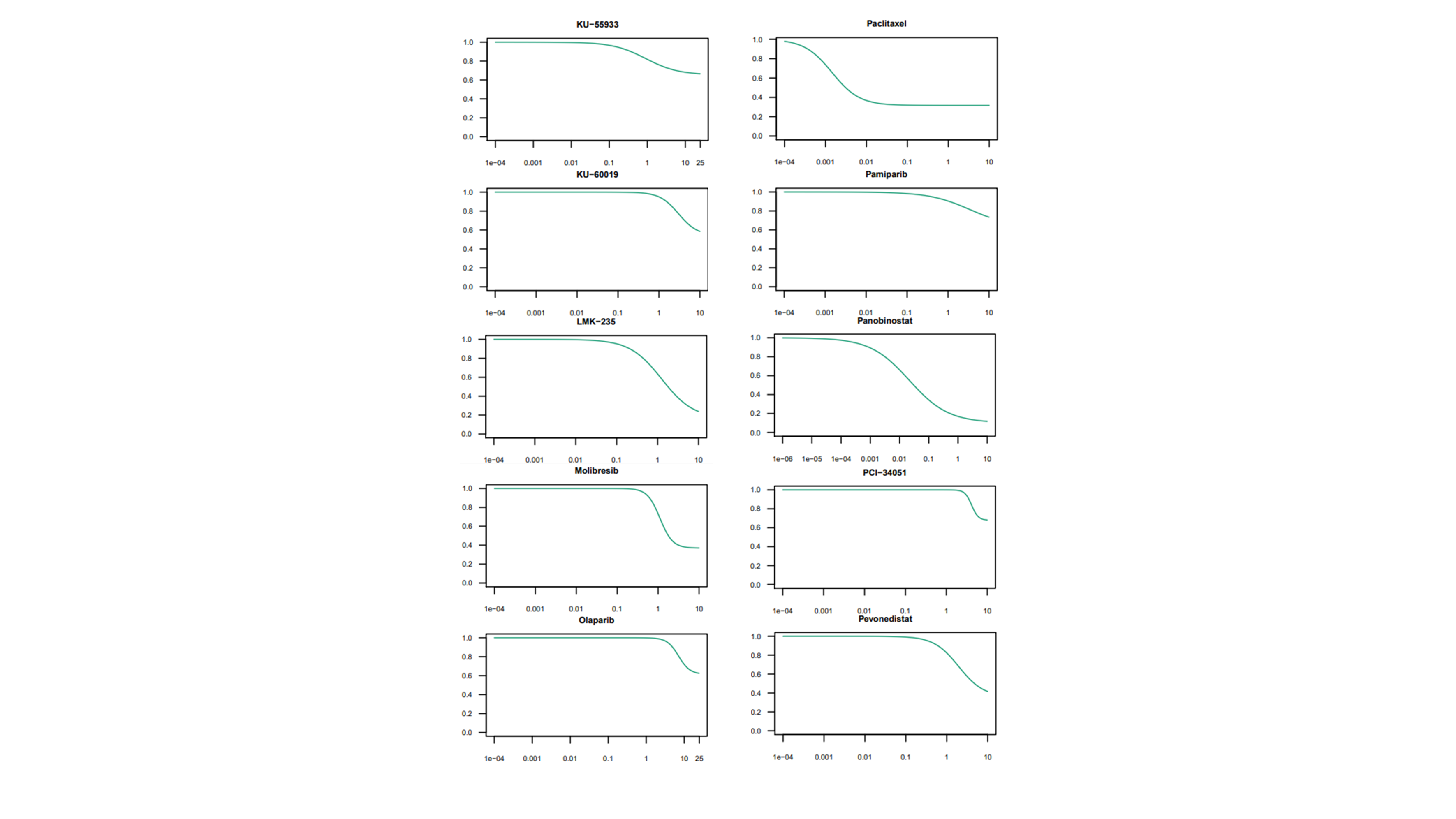


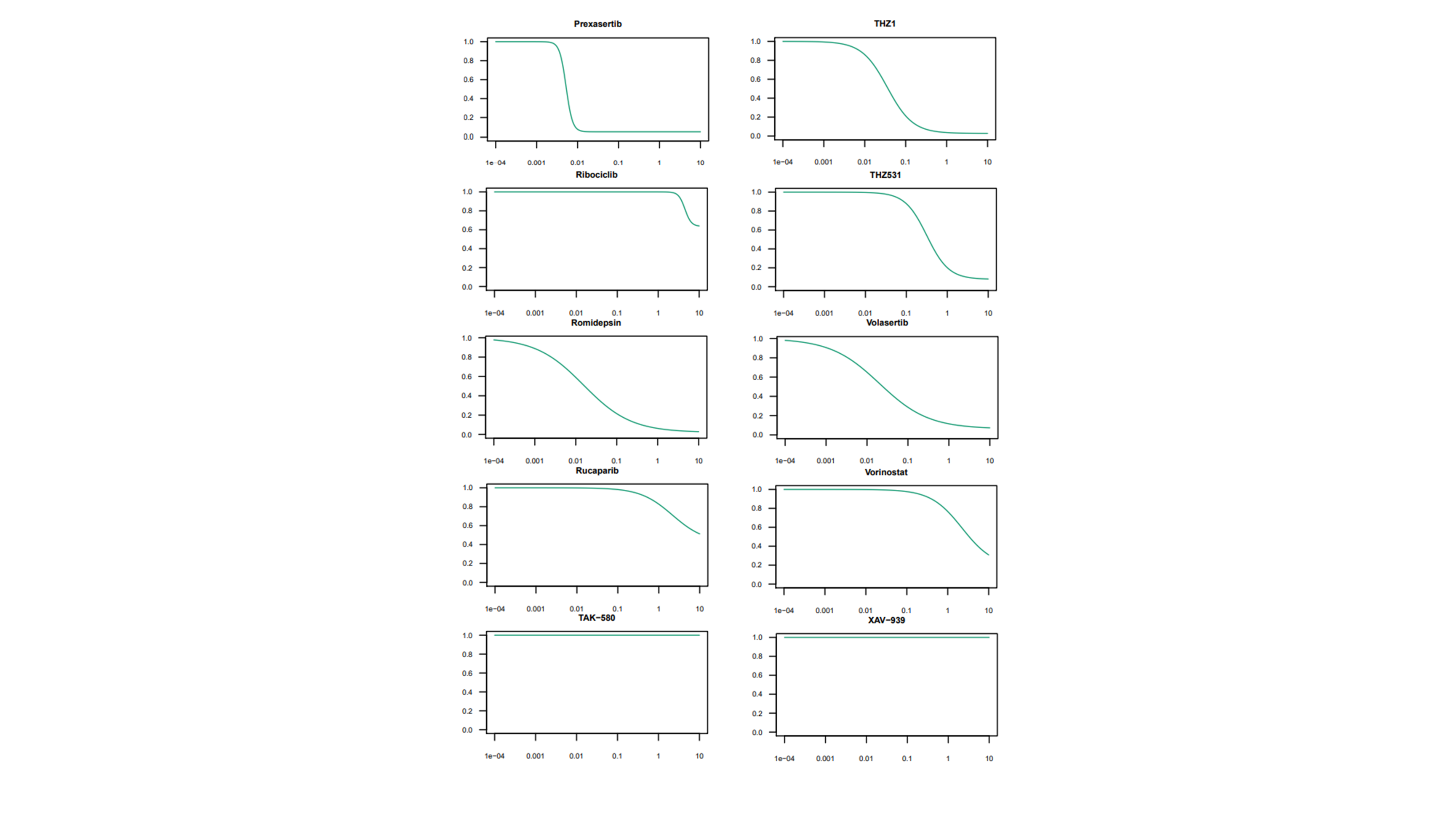


**S2 Figure: Individual MTT assay cell viability curves of the compounds used on HEK293T-eGFP cells.** IC50 calculations are summarized in Table 1. All conditions represent n=10 technical replicates.


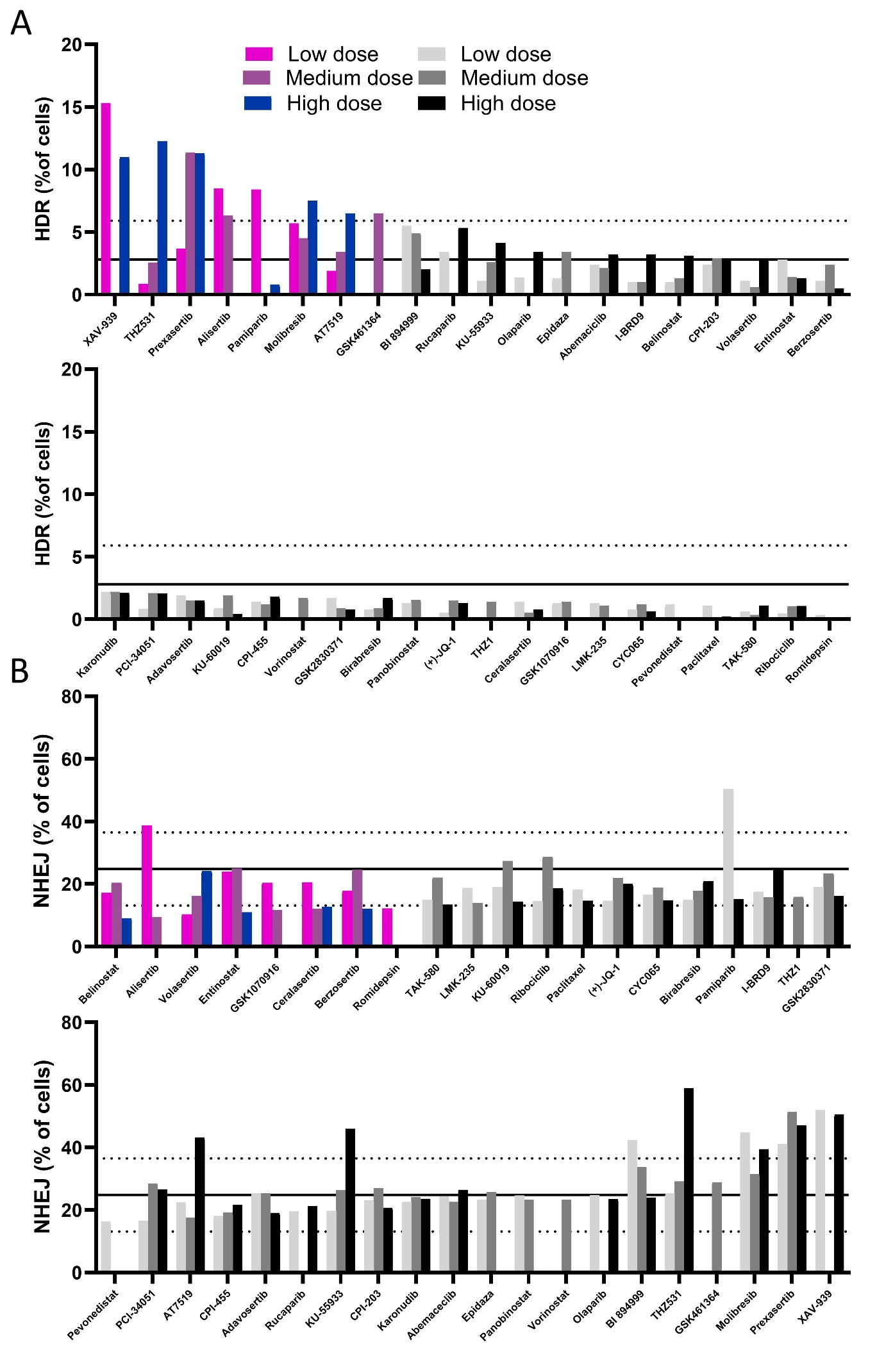


**S3 Figure: Effect of the 40 screened compounds on absolute incidence of NHEJ (A, sorted low NHEJ-high NHEJ) and HDR (B, sorted high-low HDR) compared to DMSO-treated controls (mean+- SD as solid and dotted lines, n=29 wells).** Colored hits were either lower than mean – SD for NHEJ suppression, or higher than mean + SD for HDR enhancement.


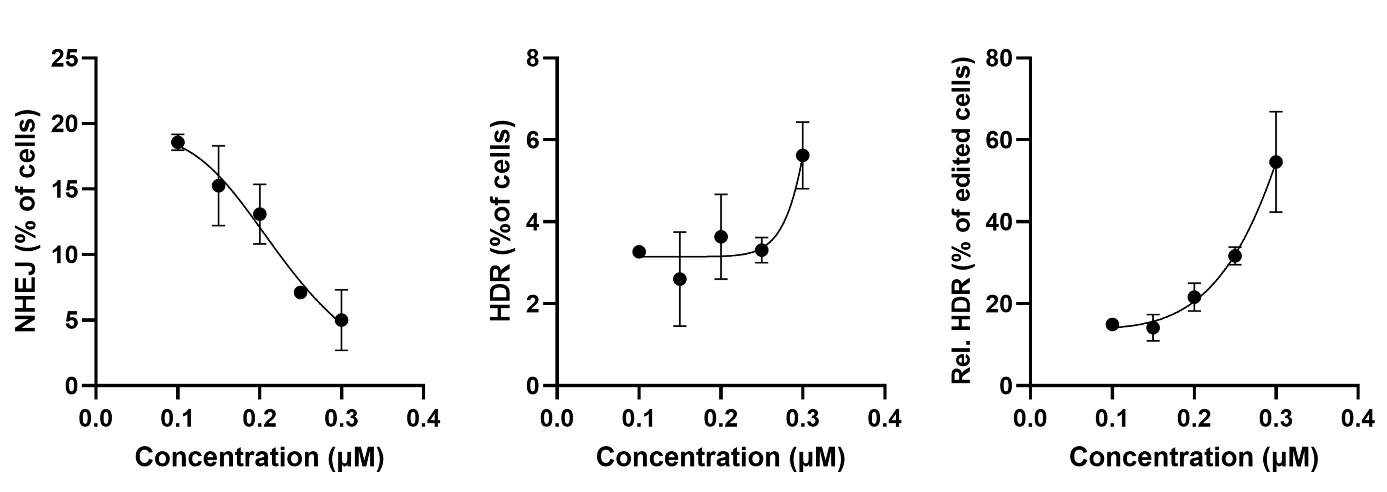


**S4 Figure: Effect of a narrow dose range of alisertib on (from left to right) NHEJ, HDR and relative HDR incidences.** N=3 technical replicates.


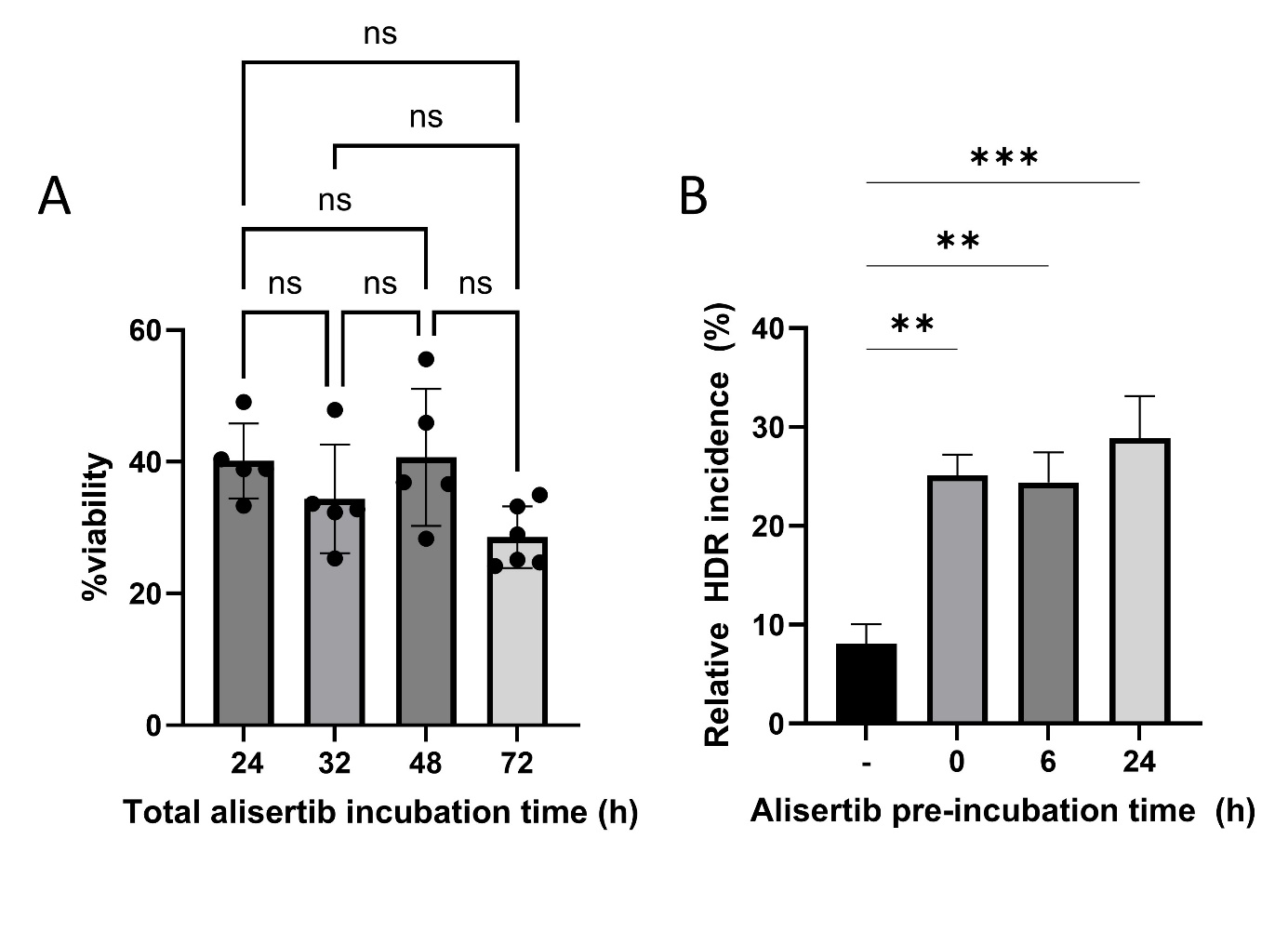


**S5 Figure: : Effect of varying incubation time with alisertib on HEK293T cells, prior to adding CRISPR formulations, on toxicity and gene editing outcomes.** A: Total alisertib incubation time was varied to assess the effect on cell viability determined by an MTS assay. Viability was calculated by subtracting the blank from all samples and dividing the measured absorption for alisertib treated cells by the absorption for vehicle-control treated cells at each timepoint. N=5 technical replicates. B: Alisertib pre-incubation (+48 hours concurrent incubation of alisertib + transfection mix) variation reveals that simultaneous incubation of alisertib and CRISPR-Cas formulations was effective, and pre-incubation with alisertib before CRISPR-Cas formulation addition was not significantly more effective than simultaneous incubation (corresponding to the 48 hours condition in panel A). n=3 technical replicates.


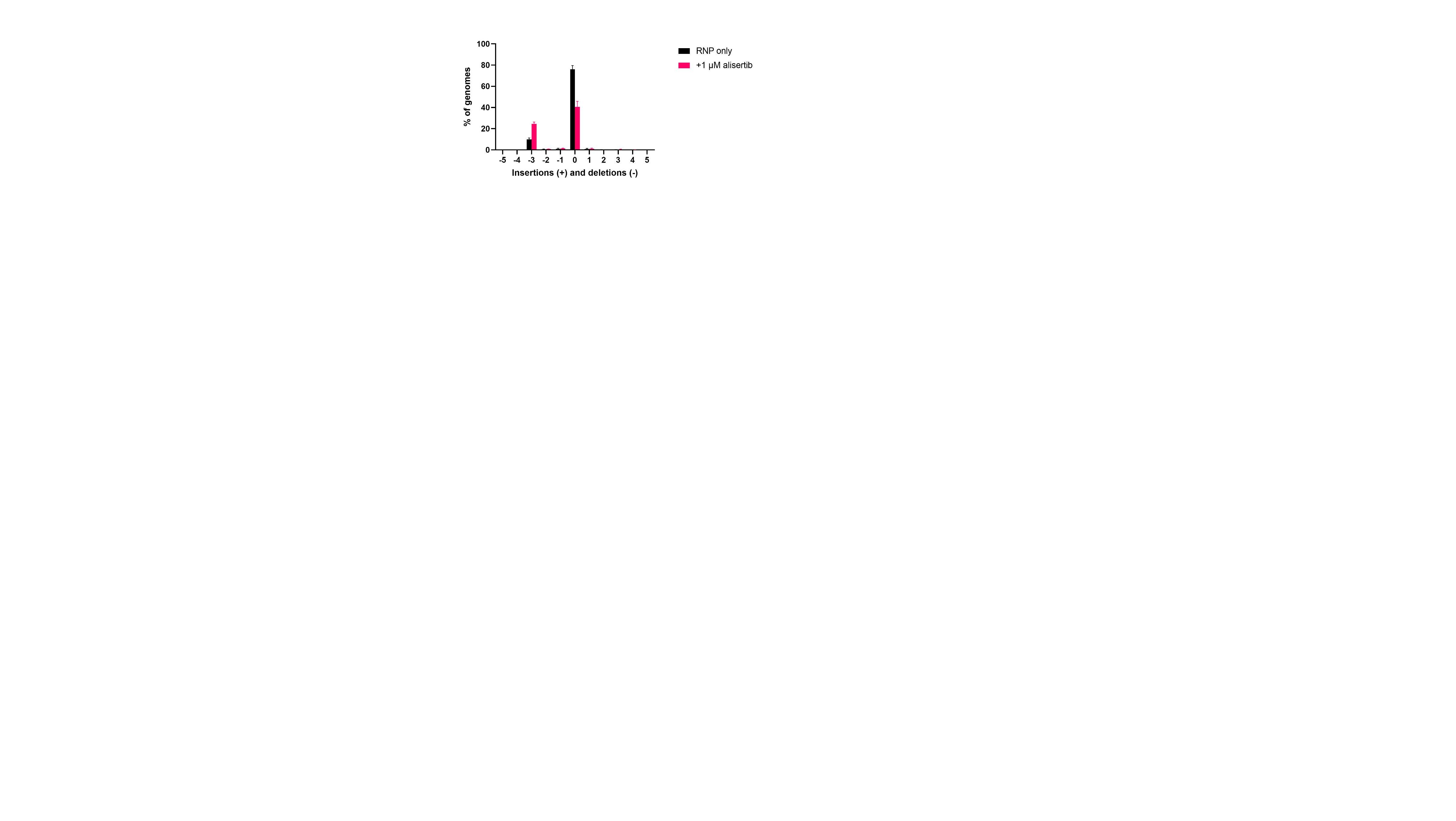


**S6 Figure: Mutation distribution in TIDER analysis, cropped at +- 5 nt.** Relatively, most mutations showed up as deletions of a whole codon (-3). N=3 technical replicates.

**S1 Method: PCSK9 genome editing in Hepa 1-6 cells**

Hepa 1-6 cells were treated with alisertib 24h prior to transfection. Transfection mixtures were prepared to deliver 30 nM RNP (and where applicable 60 nM HDR template DNA) respectively using the commercial ProdeliverIN CRISPR kit. Following this, the cells were incubated for an additional 48h followed by cell lysis and DNA purification using the DNeasy Blood and Tissue kit (Qiagen, Venlo, The Netherlands) using the manufacturers protocol for cell line lysis. Subsequently, PCR was done using the primer set in S1 Table using the Phusion Hotstart Dna Polymerase (Thermo Fischer Scientific) and the recommended thermocycle conditions and PCR mix by the manufacturer. PCR products were purified using the GeneJET PCR Purification Kit (Thermo Fischer Scientific) and finally prepared for Sanger sequencing performed by Macrogen (Amsterdam, The Netherlands. Data was analyzed using the TIDER software package available at <http://shinyapps.datacurators.nl/tider/> using an untreated sequencing plot as control chromatogram and a dsDNA gBlock (Integrated DNA Technologies) containing the desired mutation as reference chromatogram. Total gene editing and HDR were analyzed for **S7 Figure**.

An additional control was performed to validate the transfection kit, Cas9 protein and the transfection route using the methods described in the Methods section in Hepa 1-6-eGFP cells, in which eGFP knock-out was measured using flow cytometry.

**S1 Table:** **Nucleotide sequences used for PCSK9 gene editing and analysis.** The designs for the sgRNA and primers was based on previously published sequences (36).

| sgRNA | UCCGCCGUUGCUCCAAGGUA |
| --- | --- |
| ssODN template DNA | AGCATCACCCCAACCCCAAAGCAACGCCGTTGCCTGGCACGTATACTTGGAGCAACGGCGGAAGGTG  GCGGTGGCCACATG |
| PCR primer, forward | ACACCCCAGAAGGCTTCCACCT |
| PCR primer, reverse | AACCCCAACAGGCACCCAGGAT |
| gBlock containing HDR mutation | ACACCCCAGAAGGCTTCCACCTTCACGTGGACGCGCAGGCTGCCGGTGGGCTCCCGTTCTCTCT  CTCTTTCTGAGGCTAGAGGACTGAGCCAGTCCTTGGCTCCCCAGAGACATCACGGCCCGCAGC  CCCGGAGCCAAGTGCCCCGAGTCCCAGGCGTCCATGTCCTTCCCGAGGCCGCGCGCACCTCTCC  TCGCCCCGATGGGCACCCACTGCTCTGCGTGGCTGCGGTGGCCGCTGTTGCCGCTGTTGCCGCC  GCTGCTGCTGCTGTTGCTGCTACTGTGCCCCACCGGCGCTGGTGCCCAGGACGAGGATGGAGA  TTATGAAGAGCTGATGCTCGCCCTCCCGTCCCAGGAGGATGGCCTGGCTGATGAGGCCGCACA  TGTGGCCACCGCCACCTTCCGCCGTTGCTCCAAGGTATACGTGCCAGGCAACGGCGTTGCTTTG  GGGTTGGGGTGATGCTCTTCGGGGGTCTTCTCTGCTCATCTAGCCGTCTGGTGGTCTCTAAGTG  CAGCCCTGAGGTGCGGGAGGCGAGGGCAAGACTTAGTGCTCAGCTGCACCTTGTGGCACAGA  GTGATGGGGGAGGCCACGTGCTAAAGGCACTGCGGGGCTTGGTTCCAAAAGTGTGAGGCGG  GGAGCGGGCTACCAGTGTGGTCATGCAGAAAACGTGTCCTCCGAAGTAAAGTGGCATCGGG  AGGCTGAGAACTCTAGTGGCACATCTTTCTCAACTGGTCATCCAGCAGTCATCCTGGGTGCCT  GTTGGGGTT |
|  |  |


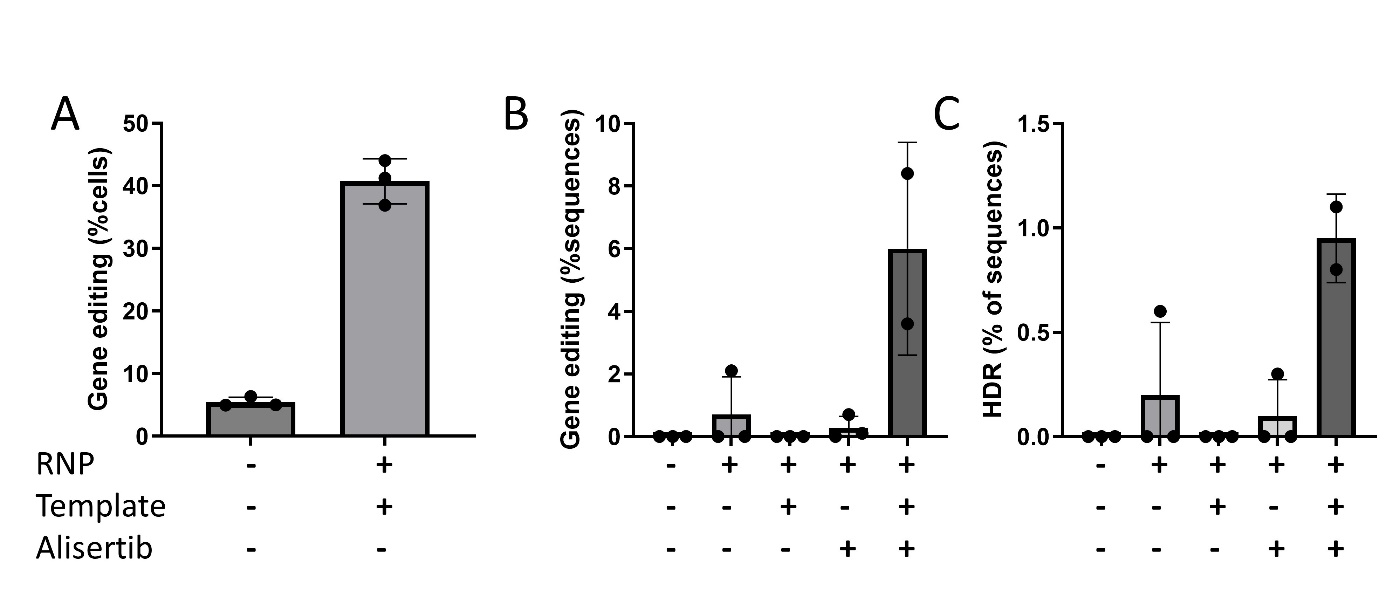


**S7 Figure:** **Efficacy of genome editing on the PCSK9 locus determined by the TIDER algorithm in Hepa 1-6 cells.** Below each bar, the used reagents are given (RNP: transfection mix containing targeted sgRNA and Cas9 protein; template: ssODN template for HDR; alisertib: 0,3 µM (Hepa 1-6) at the time of seeding). A: control experiment in Hepa 1-6-eGFP cells to validate the Cas9 transfection of Hepa 1-6 cells. Compared to the transfection mix used in B-E, only the HDR template sequence and sgRNA encoded a different sequence (given in the Methods section for eGFP and S1 Table for PCSK9). B: Total gene editing at the PCSK9 locus as determined by the TIDER algorithm in Hepa 1-6 cells. C: HDR efficiency at the PCSK9 locus as determined by the TIDER algorithm in the Hepa 1-6 cells. Each dot represents a technical replicate.
